# Editorial: Involvement of Neuro-Immune Mechanism and Brain–Gut Axis in Pathophysiology of Mood Disorders

**DOI:** 10.3389/fpsyt.2019.00403

**Published:** 2019-06-11

**Authors:** Shaohua Hu, Yiru Fang, Chee H. Ng, J. John Mann

**Affiliations:** ^1^Department of Psychiatry, First Affiliated Hospital, Zhejiang University School of Medicine, Hangzhou, China; ^2^The Key Laboratory of Mental Disorder Management of Zhejiang Province, Hangzhou, China; ^3^Division of Mood Disorders, Shanghai Mental Health Center, Shanghai Jiao Tong University School of Medicine, Shanghai, China; ^4^Department of Psychiatry, University of Melbourne, Melbourne, VIC, Australia; ^5^Department of Psychiatry, Columbia University, New York, NY, United States; ^6^Division of Molecular Imaging and Neuropathology, New York State Psychiatric Institute, Columbia University, New York, NY, United States

**Keywords:** neuro-immune, brain–gut axis, mood disorders, major depressive disorder, bipolar disorder

Mood disorders are common and generally recurrent episodic or chronic disorders that present with only depressive symptoms in the case of major depressive disorder (MDD), whereas both depressive and manic symptoms are found in bipolar disorders (BD). Mood disorders cause substantial individual, societal, and economic burden (1), and are the most common diagnosis associated with suicide. The diagnosis of mood disorders depends currently on clinical symptoms. There are no known biological markers to aid the diagnosis of mood disorders. There has been a surge of interest in the inter-relationship between neuro-immunology and gut microbiota in mood disorders. This journal issue examines the potential role of these two domains and their possible interaction in the pathogenesis of mood disorders. Pro-inflammatory cytokine elevation has been reported in mood disorders (2), and they can cross the blood–brain barrier and affect microglial activation (3). On the other hand, gut microbiota has also been associated with mood disorders (4) and can affect the brain by producing neurotransmitters and bacterial metabolites and by promoting the release of pro-inflammatory cytokines (5). Alternations of gut microbiota characteristics, especially an increase in pro-inflammatory genera, are reported in mood disorders (6). The research topic of this current journal issue provides a forum focusing on a set of investigations of the neuro-immune and brain–gut axis mechanisms in mood disorders. It includes several studies investigating potential biological markers for diagnostic and prognostic prediction in mood disorders.

Liu et al. explored the role of the kynurenine pathway in MDD. Kynurenic acid in a receiver operating characteristic (ROC) curve predicts MDD (82.5%), and area under the ROC curve remains comparable (83.6%) for an MDD diagnosis when combining kynurenic acid and quinolinic acid levels. More work is needed to determine the exact role of the kynurenine pathway in the pathogenesis of MDD, and its relevance as a potential treatment target.

A randomized controlled trial by Yang et al. assesses whether endoplasmic reticulum stress (ERS) can mediate an antidepressant effect and serve as a treatment target for depression. The chronic unpredictable mild stress (CUMS)-induced depression rat model displays depressive-like behaviors and an increase in hippocampal cell apoptosis and ERS markers glucose-regulated protein 78 and C/EBP-homologous protein. Antidepressant medications reduced the depressive-like behaviors and ERS marker levels. Therefore, ERS may be a therapeutic target for MDD. Microglia area type of macrophage in central nervous system (CNS) is involved in neuroinflammation (7).

Activated microglia increase pro-inflammatory cytokines in CNS, which in turn could decrease serotonin neurotransmission through kynurenine pathway, and affect cell apoptosis, excitotoxicity, neurogenesis, and neurotrophin production (8). The Tan et al. study employed a CUMS-induced gestating mouse model and microglia-speciﬁc autophagy-deﬁcient mice. Compared to normal mice, the microglia-speciﬁc autophagy-deﬁcient mice showed higher inﬂammatory factors and lower brain-derived neurotrophic factor (BDNF) expression levels and autophagy-associated proteins. Following 3 weeks of fluoxetine administration, depressive behavior was reversed, BDNF levels and autophagy-associated proteins increased, while inﬂammatory factors decreased.

Previous studies reported that gut microbiota affect microglial maturation and activation, as part of the brain–gut axis (9, 10). Liu et al. describe the neuro-endocrine-immune mechanisms of the brain–gut axis and summarize findings from animal and human studies that demonstrate their relationship to mood disorders. Alterations of gut microbiota characteristics are found in mood disorders, and administering prebiotics, probiotics, and suitable antibiotics could potentially reverse depressive symptoms. They conclude that although more research is needed, it may be possible to treat mood disorders by selective targeting of gut microbiota in the future.

Cheung et al. further reviewed the relationship between gut microbiota and MDD. Although statistically there were no consistent findings of gut microbiota changes in MDD in human studies, we need to refine our methodologies further to better understand how gut microbiota contribute to the pathogenesis of MDD.

Aizawa et al. focused on Biﬁdobacterium and Lactobacillus counts in patients with bipolar disorder (BD). Compared to healthy controls, no difference was found in BD regarding Biﬁdobacterium or Lactobacillus. However, negative correlations were observed between Lactobacillus counts and sleep and between Biﬁdobacterium counts and cortisol levels. These microbial taxa may not be associated with emotional symptoms, but with other disease characteristics, such as sleep and stress response. Li et al. investigated the relationship of the gut microbiota to insomnia, circadian disturbance, and depression. The authors offered a hypothesis that low-level chronic inﬂammation may be linked to sleep loss, circadian misalignment, mood disorders, and metabolic disease.

These studies provide preliminary evidence regarding the potential of biological markers of neuro-immunology and microbial markers to distinguish patients with mood disorders from healthy controls, as well as their potential as therapeutic targets. Longitudinal studies of such potential biological markers in patients will further our understanding of the cause–effect relationship between mood disorders, the gut microbiota, and neuro-immunology and their role in mediating treatment response.

## Author Contributions

SH wrote the manuscript. YF, CN, and JM edited the editorial.

## Funding

This study was funded in part by grants from the National Key Research and Development Program of China (No. 2016YFC1307104 to S.H.H.) and the Key Research Project of Zhejiang Province (No. 2015C03040 to Y.X.).

## Conflict of Interest Statement

The authors declare that the research was conducted in the absence of any commercial or financial relationships that could be construed as a potential conflict of interest.
